# Toxic Effects of Cd and Zn on the Photosynthetic Apparatus of the *Arabidopsis halleri* and *Arabidopsis arenosa* Pseudo-Metallophytes

**DOI:** 10.3389/fpls.2019.00748

**Published:** 2019-06-06

**Authors:** Michał Szopiński, Krzysztof Sitko, Żaneta Gieroń, Szymon Rusinowski, Massimiliano Corso, Christian Hermans, Nathalie Verbruggen, Eugeniusz Małkowski

**Affiliations:** ^1^Department of Plant Physiology, University of Silesia in Katowice, Katowice, Poland; ^2^Institute for Ecology of Industrial Areas, Katowice, Poland; ^3^Laboratoire de Physiologie et de Génétique Moléculaire des Plantes, Université Libre de Bruxelles, Brussels, Belgium

**Keywords:** photosystem II, Cadmium, Zinc, Arabidopsis, chlorophyll *a* fluorescence

## Abstract

Hyperaccumulation and hypertolerance of Trace Metal Elements (TME) like Cd and Zn are highly variable in pseudo-metallophytes species. In this study we compared the impact of high Cd or Zn concentration on the photosynthetic apparatus of the *Arabidopsis arenosa* and *Arabidopsis halleri* pseudo-metallophytes growing on the same contaminated site in Piekary Slaskie in southern Poland. Plants were grown in hydroponic culture for 6 weeks, and then treated with 1.0 mM Cd or 5.0 mM Zn for 5 days. Chlorophyll *a* fluorescence and pigment content were measured after 0, 1, 2, 3, 4, and 5 days in plants grown in control and exposed to Cd or Zn treatments. Moreover, the effect of TME excess on the level of oxidative stress and gas-exchange parameters were investigated. In both plant species, exposure to high Cd or Zn induced a decrease in chlorophyll and an increase in anthocyanin contents in leaves compared to the control condition. After 5 days Cd treatment, energy absorbance, trapped energy flux and the percentage of active reaction centers decreased in both species. However, the dissipated energy flux in the leaves of *A. arenosa* was smaller than in *A. halleri*. Zn treatment had more toxic effect than Cd on electron transport in *A. halleri* compared with *A. arenosa*. *A. arenosa* plants treated with Zn excess did not react as strongly as in the Cd treatment and a decrease only in electron transport flux and percentage of active reaction centers compared with control was observed. The two species showed contrasting Cd and Zn accumulation. Cd concentration was almost 3-fold higher in *A. arenosa* leaves than in *A. halleri*. On the opposite, *A. halleri* leaves contained 3-fold higher Zn concentration than *A. arenosa*. In short, our results showed that the two *Arabidopsis* metallicolous populations are resistant to high Cd or Zn concentration, however, the photosynthetic apparatus responded differently to the toxic effects.

## Introduction

Due to industrial and agricultural activities, such as mining, smelting, traffic, using of fertilizers, and sewage sludges, as well as natural processes including atmospheric deposition and weathering of minerals metal contamination has become serious environmental problem worldwide (Alloway, 2013; Su et al., 2014).

Cadmium (Cd) is considered as one of the most toxic non-essential elements for plants (Clemens and Ma, 2016). Despite zinc (Zn) being an essential microelement (Kabata-Pendias, 2011), its excess in plants can also induce phytotoxic effects (Chaney, 1993). It was found that excess of both trace metal elements (TME) can have similar negative influence on different elements of photosynthetic apparatus, for example: pigment biosynthesis, light capture, electron transport, stomatal conductance, CO_2_ assimilation, and activity of enzymes in Calvin cycle (Clijsters and Van Assche, 1985; Van Assche and Clijsters, 1986; Myśliwska-Kurdziel et al., 2002; Sagardoy et al., 2010; Vassilev et al., 2011; Verbruggen et al., 2013). However, the toxic effects of high Cd and excess Zn concentration on the complex process of photosynthesis are still poorly understood (Paunov et al., 2018).

*Arabidopsis halleri* and *A. arenosa* are pseudo-metallophytes closely related to *A. thaliana* (Clauss and Koch, 2006), which are used to study the adaptation to environments highly contaminated with TME. Both species can be commonly found on metalliferous and non-metalliferous sites in southern Poland (Fiałkiewicz and Rostanski, 2006; Szarek-Łukaszewska and Grodzinska, 2007, 2011; Preite et al., 2018). It was documented that metalliferous populations of *A. halleri* and *A. arenosa* are hypertolerant to Cd and Zn (Przedpełska and Wierzbicka, 2007; Nadgórska-Socha et al., 2013; Sitko et al., 2017; Stein et al., 2017). While Zn hyperaccumulation is a constitutive trait, Cd accumulation is highly variable within *A. halleri* populations (Pauwels et al., 2012; Meyer et al., 2015; Sitko et al., 2017; Stein et al., 2017; Corso et al., 2018; Frérot et al., 2018; Schvartzman et al., 2018), whereas these traits are poorly studied in *A. arenosa*.

Measurements of chlorophyll *a* fluorescence is a noninvasive and sensitive method for monitoring physiological status of plants (Baker, 2008; Kalaji et al., 2012, 2014; Sitko et al., 2017). In recent years such measurements have become more and more commonly used under various conditions such as TME stress (Kalaji et al., 2014; Daszkowska-Golec et al., 2017; Sitko et al., 2017; Paunov et al., 2018). Analysis of chlorophyll *a* fluorescence kinetics, known as OJIP transient, can provide information on electron transport reactions mainly inside PSII and parts of PSI (Baker, 2008; Kalaji et al., 2014; Goltsev et al., 2016; Paunov et al., 2018). In order to obtain more complete picture of effect of TME excess on photosynthesis, measurements of leaf gas-exchange parameters such as photosynthetic rate based on CO_2_ assimilation and parameters based on chlorophyll *a* fluorescence are necessary (Arshad et al., 2015; Rusinowski et al., 2019).

Measurements of pigment contents such as chlorophyll, flavonols and anthocyanins have also become increasingly popular due to the development of portable devices, which enable measurements of these pigment contents in laboratory as well as *in situ* (Cerovic et al., 2015; Lefebvre et al., 2016; Gonzalez-Mendoza et al., 2017; Hosseini et al., 2017; Sitko et al., 2017). Flavonols and anthocyanins belong to flavonoid secondary metabolites, which are the largest class of polyphenols (around 8,000 metabolites) in plants (Mattivi et al., 2006; Tohge et al., 2017). Flavonoids are characterized by two benzene rings linked by a heterocyclic pyran ring and primarily occurring in plants as O-glycosides. They play a major role in plant protection against negative effects of abiotic and biotic stress factors in model and crop plants (Jaakola et al., 2004; Corso et al., 2015; Tohge et al., 2017). Flavonols and anthocyanins can serve as protection against damage caused by TME, free oxygen radicals and excessive light radiation (Gould, 2004; Emiliania et al., 2013; Landi et al., 2015; Peng et al., 2017; Tohge et al., 2017; Moustaka et al., 2018).

High concentrations of TME such as Cd and Zn can interfere with numerous physiological processes in plants. As a result the overproduction of reactive oxygen species (ROS) such as H_2_O_2_ occurs and oxidative stress in plants is observed (Sandalio et al., 2009; Moura et al., 2012). One of the enzymes, which is responsible for the decomposition of H_2_O_2_ is catalase. For this reason, the changes in activity of this enzyme are used as a marker of plant resistance to oxidative stress (Moura et al., 2012; Rusinowski et al., 2019). When excess of ROS and/or H_2_O_2_ appeared in plants and oxidative stress develops, lipid peroxidation takes place. One of the products of lipid degradation is malondialdehyde (MDA). Thus, the increase in the content of this chemical compound in plant tissues is frequently taken into account as a measure of oxidative stress level (Bouazizi et al., 2010; Rusinowski et al., 2019).

The aim of this study was to compare the response of the photosynthetic apparatus of two hypertolerant plant species to high concentration of Cd or Zn living on the same contaminated site. In this work we compared physiological responses of two metallicolous *A. arenosa* and *A. halleri* populations from the contaminated site of Piekary Slaskie in South Poland. Presented in the current paper results for chlorophyll *a* fluorescence indicate that *A. arenosa* shows similar tolerance to Cd and Zn as *A. halleri*.

## Materials and Methods

### Plant Material and Culture Conditions

Plants of *Arabidopsis halleri* and *Arabidopsis arenosa* were grown hydroponically in controlled greenhouse conditions. Seeds of both populations were collected from the same metalliferous site in Piekary Slaskie (50°22′00.6“N, 18°58′18.4”E). Vernalized seeds were sown onto vermiculite watered with deionized water for the first 2 weeks and for the next 2 weeks with 1/10 strength Hoagland solution. Four-week-old seedlings were transferred into hydroponic containers (three seedlings of each species per container) filled with 2 L of ½ strength Hoagland solution (Bloom, 2006) with initial pH adjusted to 5.8 ± 0.05. Seedlings were grown in a greenhouse under artificial light with high pressure sodium lamps (HPS), photoperiod 12 h light (150 μE m^−2^ s^−1^)/12 h darkness. The temperature in the greenhouse was 20 ± 1°C and air humidity was 60 ± 5%. The medium was changed twice a week. Plants were grown for 6 weeks in the control solution. After 6 weeks of growth containers were divided into 3 experimental groups: Control (2 containers, 6 plants per species), Cd treatment (3 containers, 9 plants per species), and Zn treatment (3 containers, 9 plants per species). Cd was added at the concentration of 1,000 μM and Zn at the concentration of 5,000 μM, two concentrations that are lethal for non-tolerant plant species, as *Arabidopsis thaliana*. These concentrations were chosen in order to induce toxic effect on photosynthetic apparatus. The concentrations were chosen on the basis of preliminary studies, where half of mentioned concentrations did not show toxic effect on photosynthetic apparatus performance in short-term experiment (data not shown). The experiment was finished after 5 days and all measurements were made after 0, 1, 2, 3, 4, and 5 days, except growth parameters (shoot and root fresh weight, and root length) and accumulation of TME, which was performed on plant material harvested at the end of experiment. The experiment was repeated three times.

### Measurements of Plant Growth

At the end of the experiment, plants were harvested and root length was measured. Afterwards, shoot and root fresh biomasses were measured separately.

### Analysis of Cd and Zn Accumulation in Leaves

At the end of the experiment leaves of plants were harvested and dried at 80°C for 72 h. Dry plant material was ground in the mortar and subsequently digested in a microwave-assisted wet digestion system (ETHOS 1, Milestone, Italy) according to the procedure provided by the manufacturer (concentrated HNO_3_ and 30% H_2_O_2_, 4:1 v/v). The Cd and Zn concentrations in leaves were analyzed in the digests using flame atomic absorption spectrophotometer (iCE 3,500 FAAS, Thermo Scientific, USA). Reference plant material (Oriental Basma Tobacco Leaves (INCT-OBTL-5), Institute of Nuclear Chemistry and Technology, Poland) was used for the quality assurance of the analytical data.

### Measurements of Chlorophyll *a* Fluorescence and Pigment Index

Measurements were done on fully developed *Arabidopsis halleri* and *A. arenosa* leaves which entirely filled the area of the sensor. Chlorophyll *a* fluorescence was measured at 0, 1, 2, 3, 4, and 5 days using the Plant Efficiency Analyzer (PocketPEA fluorimeter, Hansatech Instruments Ltd., England). Before measurement, each selected leaf was adapted in the dark for 30 min using dedicated leaf clips. After adaptation, a saturating light pulse of 3,500 μE m^−2^ s^−1^ was applied for 1 s, which closed all the reaction centers, and the fluorescence parameters were measured. Measurements were done without damaging the plant material.

Chlorophyll, flavonol, and anthocyanin index were measured with the use of Dualex Scientific+ sensor (Force-A, France). Measurements of pigment index were performed at 0, 1, 2, 3, 4, and 5 days on the same leaves as for chlorophyll *a* fluorescence measurements. Measurements were done without damaging the plant material.

### Gas-Exchange Parameters

Plant gas exchange parameters, such as intracellular CO_2_ concentration (*Ci*), photosynthetic rate (*A*), stomatal conductance (*g*_*s*_), and transpiration rate (*E*) were conducted on fully developed leaves. Measurements were carried out at the end of experiment, after 5 days using an infrared gas analyzer with special chamber for *Arabidopsis* (LCpro+, ADC Bioscientific, UK) under controlled climate conditions (*T* = 24°C, Ambient light PAR = 150 μmol m^−2^ s^−1^). Measurements were performed at noon.

### Oxidative Stress Parameters

Hydrogen peroxide concentration was determined as described by Bouazizi et al. (2010) with minor modifications. Fresh leaf tissues (150 mg) were homogenized in 1.5 ml of 0.1% trichloroacetic acid (TCA). The homogenate was centrifuged at 12,000 *g* for 15 min and 0.5 ml of the supernatant was added to 0.5 ml potassium phosphate buffer (10 mM, pH 7.0) and 1 ml potassium iodide (KI) (1 M). The absorbance of the supernatant was measured at 390 nm, and the content of H_2_O_2_ was obtained using a standard curve.

The level of lipid peroxidation estimated by malondialdehyde (MDA) concentration was determined as described by Bouazizi et al. (2010) with minor modifications. Fresh leaf tissues (150 mg) were homogenized in 4 ml 0.25% thiobarbituric acid (TBA) prepared in 10% TCA. The homogenate was incubated in a water bath at 95°C for 30 min and then cooled in an ice bath. After centrifugation at 10,000 *g* for 10 min, the absorbance of the supernatant was measured at 532 nm and corrected by subtracting the non-specific absorbance at 600 nm. Concentration of malondialdehyde (MDA) was calculated using an extinction coefficient of 155 mM^−1^ cm^−1^.

The activity of catalase was determined as described by Bouazizi et al. (2010) with minor modifications. Fresh leaf tissues (150 mg) were homogenized in 1.5 ml potassium phosphate buffer (10 mM, pH 7.0). The homogenate was centrifuged at 12,000 *g* for 20 min. The supernatant (0.02 ml) was added to 2 ml 10 mM peroxide prepared in potassium phosphate buffer. Catalase activity was determined spectrophotometrically by monitoring the changes of absorbance caused by H_2_O_2_ reduction at 240 nm.

### Statistical Analysis

The statistically significant differences among mean values were determined using one-way ANOVA and *post hoc* Tukey HSD test (*P* < 0.05). Additionally two-way ANOVA analysis was performed to determine effect of treatment (Ctr, 1.0 mM Cd and 5.0 mM Zn), species (*A. arenosa* and *A. halleri*) as well as interaction between these two factors on measured physiological parameters (Table S1). The statistical analysis was performed using the computer software Statistica v.13.1 (Dell Inc., USA). The pipeline models of energy fluxes through leaf's cross section were done using CorelDRAW X6 (Corel Corp., Canada).

## Results

### Cd and Zn Content in *A. halleri* and *A. arenosa* Leaves

**Figure 1:**
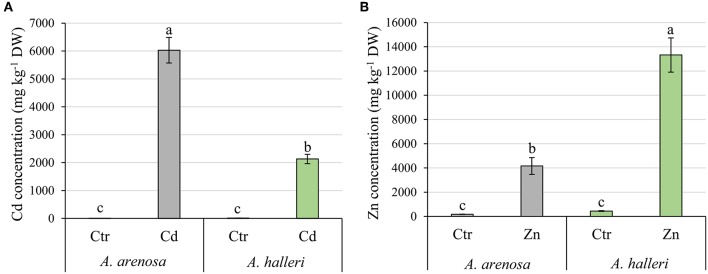
Accumulation of Cd **(A)** and Zn **(B)** in *A. arenosa* (gray) and *A. halleri* (green) leaves after 120 h of experiment. Ctr—control; Cd−1.0 mM Cd treatment; Zn−5.0 mM Zn treatment. Values are means ± SE (*n* = 6). Means followed by the same letter for each metal treatment are not significantly different from each other using the HSD test (*P* < 0.05).

Cadmium and zinc concentrations were measured in leaves of both plant species (Figure 1). Remarkably, *A. arenosa* accumulated 3-fold more Cd in the leaves with respect to *A. halleri* (6 and 2 g Cd kg^−1^ DW in *A. arenosa* and *A. halleri*, respectively) (Figure 1A). In contrast, *A. halleri* leaves accumulated 13 g Zn kg^−1^ DW, which was 3-fold more compared to Zn accumulated by *A. arenosa* (Figure 1B).

### Plant Growth

**Figure 2:**
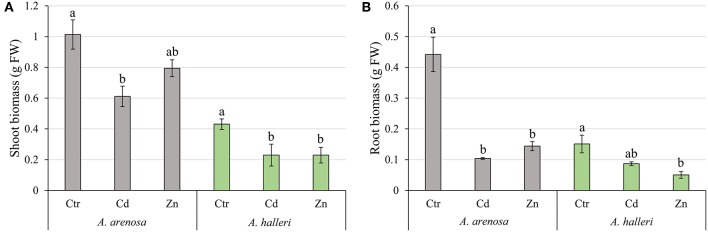
Growth of the shoots **(A)** and roots **(B)** of *A. arenosa* (gray) and *A. halleri* (green) after 120 h of experiment. Ctr—control; Cd−1.0 mM of Cd treatment; Zn−5.0 mM of Zn treatment. Values are means ± SE (*n* = 6). Means followed by the same letter for each species are not significantly different from each other using the HSD test (*P* < 0.05).

In our control conditions *A. arenosa* plants grew better than *A. halleri*, with a biomass over 2-fold higher than *A. halleri* (Figures 2A,B). The biomass of *A. arenosa* shoots was more affected by Cd treatment (60% of control) than by Zn (78% of the control), whereas the biomass of *A. halleri* shoots was similarly affected, irrespective of the metal (53% of the control) (Figure 2A). The root biomass in Cd treatment was significantly lower only in *A. arenosa* (23% of the control), whereas Zn treatment considerably diminished root growth (33% of the control) of both species (Figure 2B). Only plants of *A. arenosa* had their root length significantly lowered (73% of the control) under Zn treatment (Figure S1). Pictures of plants at the end of experiment are presented in Figure S2. Two-way ANOVA analysis showed that both treatment type and species significantly influenced shoot and root biomass, however, interaction between treatment type and species was significant only for root biomass (Table S1).

### Oxidative Stress

**Figure 3:**
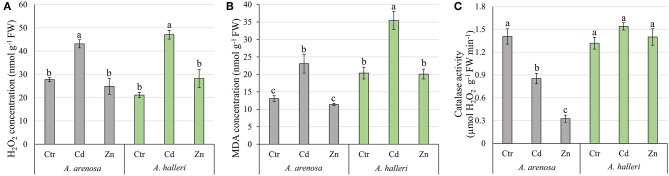
Concentration of H_2_O_2_ **(A)**, MDA **(B)**, and catalase activity **(C)** in shoots of *A. arenosa* (gray) and *A. halleri* (green) after 120 h of experiment. Ctr—control; Cd−1.0 mM Cd treatment; Zn−5.0 mM Zn treatment. Values are means ± SE (*n* = 6). Means followed by the same letter are not significantly different from each other using the HSD test (*P* < 0.05).

Concentrations of H_2_O_2_ and MDA were significantly increased by Cd treatment in leaves of both species, but not Zn (Figures 3A,B). The treatment type had significant impact on both H_2_O_2_ and MDA concentration, whereas species had significant impact only on MDA concentration. There was no significant interaction between species and treatment for both parameters (Table S1).

Catalase activity was lowered by Cd and Zn treatments in leaves of *A. arenosa* compared with control, but not in in *A. halleri* (Figure 3C). Both factors (species and treatment) had significant influence on catalase activity, moreover effect of treatment was dependent on the species (Table S1).

### Pigment Indices

**Figure 4:**
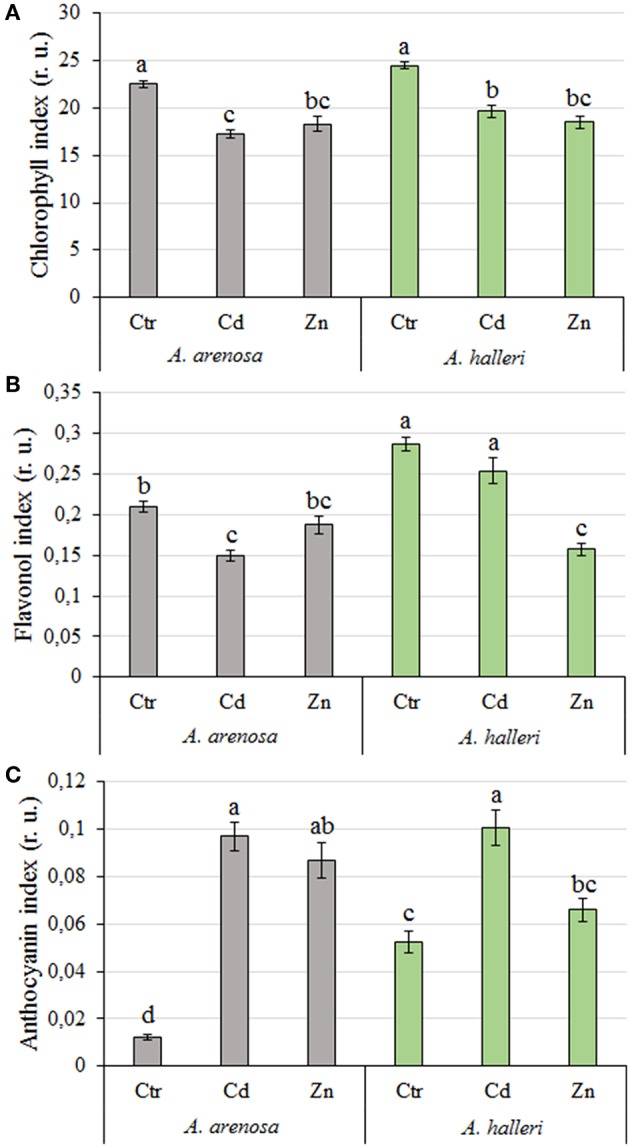
Chlorophyll **(A)**, flavonol **(B)**, and anthocyanin **(C)** index (relative units) in *A. arenosa* (gray) and *A. halleri* (green) leaves after 120 h of experiment. Ctr—control; Cd−1.0 mM Cd treatment; Zn−5.0 mM Zn treatment. Values are means (*n* = 30). Means followed by the same letter are not significantly different from each other, using Tukey HSD test (*P* < 0.05).

The chlorophyll index in *A. arenosa* and *A. halleri* was similar after 5 days Cd or Zn treatments compared to their respective controls (Figure 4A).

The flavonol and anthocyanin index was significantly higher in leaves of *A. halleri* compared to *A. arenosa* in control conditions (Figures 4B,C). After 5 days Cd treatment the flavonol index substantially decreased in leaves of *A. arenosa* compared to the control, which was not observed in *A. halleri*. In marked contrast, Zn treatment did not affect the flavonol index of *A. arenosa* leaves, whereas it decreased by 50% the one of *A. halleri* in comparison to the control (Figure 4B). Five days Cd treatment caused similar significant increase in the anthocyanin index of *A. arenosa* and *A. halleri* leaves compared with the control, whereas anthocyanin index in Zn treated plants was increased only in *A. arenosa* (Figure 4C).

Both factors (species and treatment) had significant influence on chlorophyll and flavonol indices, whereas anthocyanin index was differentiated only by treatment (Table S1).

Changes over time in the chlorophyll, flavonol and anthocyanin contents during 5 days treatments are presented in supplementary material (Figure S3).

### Photosynthetic Apparatus Performance

**Figure 5:**
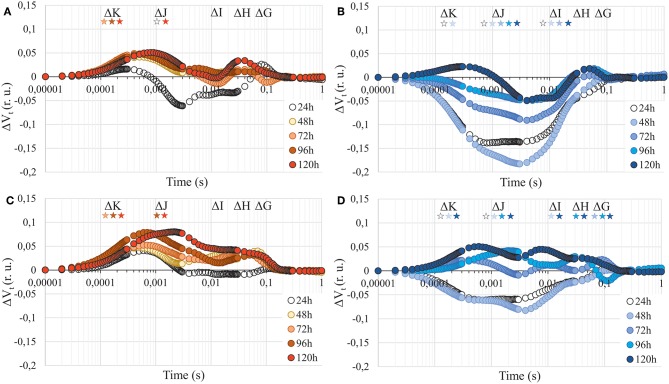
Chlorophyll *a* fluorescence induction curves presented as relative variable fluorescence (ΔV_t_) after 24, 48, 72, 96, and 120 h of experiment. **(A)**—*A. arenosa* treated with 1.0 mM Cd, **(B)**—*A. halleri* treated with 1.0 mM Cd, **(C)**—*A. arenosa* treated with 5.0 mM Zn, **(D)**—*A. halleri* treated with 5.0 mM Zn. For ΔV_t_ analysis, the fluorescence of control was the reference and equaled 0. Values are means (*n* = 8). Asterisks indicate statistically significant differences (Fisher's LSD test, *P* < 0.05) between fluorescence of plants treated with HM and the control for each band (ΔK, ΔJ, ΔI, ΔH, and ΔG). Asterisk are arranged chronologically from 24 to 120 h and asterisks color corresponds to point color, which represent the time of metal treatment. (r. u.), relative units.

The parameters describing photosynthesis performance are listed in Table S2. Photosynthetic apparatus performance was differently affected by Cd in *A. arenosa* and *A. halleri* (Figures 5A,B). Minimal fluorescence (F_0_) gradually increased in *A. halleri* treated with Cd, whereas it was not affected in *A*. *arenosa* in the same conditions (Table 1). The presence of the positive ΔK-band in ΔV_t_ curves in *A. arenosa* may be correlated with damage or uncoupling of Oxygen Evolving Complex (OEC) caused by Cd treatment (Figure 5A). Toxic effect of Cd in point ΔJ was observed in *A. arenosa* after 48 h of treatment and it remained unchanged till the end of experiment, whereas no significant changes were observed for ΔI compared with the control. Changes observed between ΔJ-ΔI can be attributed to the reduced rate of electron transfer between quinone acceptors (from Q_A_ to Q_B_). Slight increase in values compared with the control in points ΔH and ΔG observed in *A. arenosa* treated with Cd can be connected with damage to plastoquinone pool and PSI end of electron acceptors such as Ferredoxin-NADP^+^ Reductase (FNR). In contrast to *A. arenosa*, Cd seemed to stimulate performance of photosynthetic apparatus of *A. halleri* compared with control, with slight negative effect observed only after 5 days in points ΔK and ΔH (Figure 5B). Maximal fluorescence (F_m_) in *A. arenosa* gradually decreased during Cd exposure and was significantly lower after 4 days, whereas F_m_ in *A. halleri* significantly decreased only after 5 days (Table 1). Zn treatment affected differently *A. arenosa* and *A. halleri* performance of photosynthetic apparatus. Minimal fluorescence (F_0_) was not affected by Zn treatment in *A. arenosa* (Table 1), whereas in *A. halleri* F_0_ increased considerably with the exposure to high concentration of Zn (Table 1). *A. arenosa* was more affected by Zn treatment compared with *A. halleri* (Figures 5C,D). Toxic effect of Zn excess in *A. arenosa* compared with control was observed earlier than in *A. halleri*. Moreover, in *A. halleri* at the beginning of Zn treatment slight stimulation of photosynthetic apparatus was observed and at the end of the experiment the toxic effect was not as high as in *A. arenosa* (Figures 5C,D). F_m_ was significantly lowered in *A. arenosa* after 5 days of Zn treatment, whereas in *A. halleri* this parameter was not affected by the treatment (Table 1).

**Table 1:** Characteristics of the photosynthetic apparatus.

**Treatment**	**Species**	**Time (h)**	**F_**0**_**	**F_**m**_**	**ϕD_**0**_**	**ϕP_**0**_**	**ΨE_**0**_**	**ϕE_**0**_**	**δR_**0**_**	**ϕR_**0**_**
**Control**	***A. arenosa***	0	5650 ± 160*ab*	30500 ± 370*b*	0.18 ± 0.00*ab*	0.82 ± 0.00*ab*	0.47 ± 0.01*ab*	0.38 ± 0.01*ab*	0.32 ± 0.02*a*	0.12 ± 0.01*a*
		24	6470 ± 430*a*	32900 ± 650*ab*	0.20 ± 0.01*a*	0.80 ± 0.01*b*	0.44 ± 0.02*b*	0.35 ± 0.02*b*	0.28 ± 0.02*a*	0.10 ± 0.01*a*
		48	5650 ± 60*ab*	32300 ± 350*ab*	0.17 ± 0.00*ab*	0.83 ± 0.00*ab*	0.50 ± 0.01*ab*	0.41 ± 0.01*ab*	0.32 ± 0.01*a*	0.13 ± 0.01*a*
		72	5640 ± 140*ab*	32100 ± 400*ab*	0.18 ± 0.00*ab*	0.82 ± 0.00*ab*	0.48 ± 0.01*ab*	0.40 ± 0.01*ab*	0.32 ± 0.01*a*	0.13 ± 0.01*a*
		96	5660 ± 110*ab*	33100 ± 650*a*	0.17 ± 0.00*b*	0.83 ± 0.00*a*	0.51 ± 0.01*a*	0.43 ± 0.01*a*	0.29 ± 0.01*a*	0.12 ± 0.01*a*
		120	5270 ± 120*b*	31000 ± 760*ab*	0.17 ± 0.00*b*	0.83 ± 0.00*a*	0.50 ± 0.02*ab*	0.41 ± 0.01*ab*	0.32 ± 0.01*a*	0.13 ± 0.01*a*
	***A. halleri***	0	6580 ± 390*b*	30800 ± 490*a*	0.21 ± 0.01*b*	0.79 ± 0.01*a*	0.34 ± 0.02*b*	0.27 ± 0.02*ab*	0.30 ± 0.01*ab*	0.08 ± 0.01*ab*
		24	7130 ± 500*a*	31000 ± 720*a*	0.26 ± 0.02*a*	0.74 ± 0.02*b*	0.28 ± 0.03*b*	0.21 ± 0.03*b*	0.25 ± 0.02*c*	0.06 ± 0.01*b*
		48	6900 ± 270*ab*	32100 ± 610*a*	0.22 ± 0.01*b*	0.78 ± 0.01*a*	0.35 ± 0.02*b*	0.27 ± 0.02*ab*	0.29 ± 0.01*b*	0.08 ± 0.01*ab*
		72	6440 ± 190*b*	32400 ± 560*a*	0.20 ± 0.01*b*	0.80 ± 0.01*a*	0.37 ± 0.01*b*	0.30 ± 0.01*ab*	0.30 ± 0.01*ab*	0.09 ± 0.00*a*
		96	6120 ± 190*b*	32700 ± 620*a*	0.19 ± 0.00*b*	0.81 ± 0.00*a*	0.43 ± 0.01*a*	0.35 ± 0.01*a*	0.31 ± 0.01*a*	0.11 ± 0.01*a*
		120	5970 ± 140*b*	31700 ± 720*a*	0.19 ± 0.00*b*	0.81 ± 0.00*a*	0.40 ± 0.01*ab*	0.33 ± 0.01*a*	0.32 ± 0.01*a*	0.10 ± 0.01*a*
**Cd**	***A. arenosa***	0	5690 ± 230*a*	31400 ± 1000*a*	0.18 ± 0.00*b*	0.82 ± 0.00*a*	0.55 ± 0.03*a*	0.45 ± 0.02*a*	0.29 ± 0.01*a*	0.13 ± 0.00*a*
		24	5580 ± 240*a*	31800 ± 600*a*	0.18 ± 0.01*b*	0.82 ± 0.01*a*	0.48 ± 0.02*a*	0.40 ± 0.02*a*	0.28 ± 0.01*ab*	0.11 ± 0.00*ab*
		48	6150 ± 360*a*	30300 ± 500*ab*	0.20 ± 0.01*b*	0.80 ± 0.01*ab*	0.48 ± 0.02*a*	0.38 ± 0.02*a*	0.27 ± 0.01*ab*	0.11 ± 0.01*ab*
		72	5600 ± 300*a*	29600 ± 550*ab*	0.19 ± 0.01*b*	0.81 ± 0.01*ab*	0.48 ± 0.01*a*	0.39 ± 0.02*a*	0.25 ± 0.01*b*	0.10 ± 0.01*bc*
		96	5860 ± 400*a*	29000 ± 630*b*	0.20 ± 0.01*ab*	0.80 ± 0.01*b*	0.47 ± 0.02*a*	0.38 ± 0.02*a*	0.25 ± 0.01*b*	0.09 ± 0.01*bc*
		120	6590 ± 540*a*	27400 ± 1360*b*	0.25 ± 0.03*a*	0.75 ± 0.03*b*	0.47 ± 0.03*a*	0.36 ± 0.02*a*	0.22 ± 0.02*b*	0.08 ± 0.01*c*
	***A. halleri***	0	6130 ± 150*b*	33000 ± 480*a*	0.19 ± 0.00*c*	0.81 ± 0.00*a*	0.55 ± 0.02*ab*	0.45 ± 0.02*ab*	0.30 ± 0.02*ab*	0.13 ± 0.01*a*
		24	5960 ± 70*c*	34800 ± 980*a*	0.17 ± 0.00*c*	0.83 ± 0.00*a*	0.46 ± 0.02*abc*	0.38 ± 0.01*bc*	0.30 ± 0.01*ab*	0.11 ± 0.01*a*
		48	7030 ± 430*b*	32900 ± 320*a*	0.21 ± 0.01*bc*	0.79 ± 0.02*ab*	0.44 ± 0.02*abc*	0.35 ± 0.02*bc*	0.26 ± 0.01*bc*	0.09 ± 0.01*ab*
		72	8110 ± 530*a*	33200 ± 590*a*	0.24 ± 0.02*b*	0.76 ± 0.02*bc*	0.36 ± 0.04*c*	0.28 ± 0.03*c*	0.20 ± 0.02*c*	0.06 ± 0.01*b*
		96	7980 ± 560*ab*	32300 ± 650*ab*	0.25 ± 0.02*ab*	0.75 ± 0.02*bc*	0.38 ± 0.04*c*	0.29 ± 0.04*c*	0.21 ± 0.02*c*	0.07 ± 0.01*b*
		120	7850 ± 550*ab*	29700 ± 590*b*	0.27 ± 0.02*ab*	0.73 ± 0.02*c*	0.45 ± 0.03*bc*	0.34 ± 0.03*bc*	0.22 ± 0.02*bc*	0.08 ± 0.01*b*
**Zn**	***A. arenosa***	0	6420 ± 250*a*	34400 ± 870*a*	0.19 ± 0.01*a*	0.81 ± 0.01*a*	0.58 ± 0.02*a*	0.47 ± 0.01*a*	0.32 ± 0.02*a*	0.15 ± 0.01*a*
		24	5610 ± 190*a*	31400 ± 500*ab*	0.18 ± 0.01*a*	0.82 ± 0.01*a*	0.49 ± 0.01*b*	0.40 ± 0.01*b*	0.28 ± 0.01*a*	0.11 ± 0.01*b*
		48	6140 ± 180*a*	30400 ± 340*b*	0.20 ± 0.01*a*	0.80 ± 0.01*a*	0.49 ± 0.02*b*	0.39 ± 0.01*b*	0.28 ± 0.02*a*	0.11 ± 0.01*b*
		72	6180 ± 300*a*	32400 ± 780*ab*	0.19 ± 0.01*a*	0.81 ± 0.01*a*	0.46 ± 0.02*b*	0.38 ± 0.02*b*	0.26 ± 0.01*a*	0.10 ± 0.01*b*
		96	6150 ± 340*a*	32000 ± 750*ab*	0.19 ± 0.01*a*	0.81 ± 0.01*a*	0.46 ± 0.02*b*	0.37 ± 0.02*b*	0.26 ± 0.01*a*	0.10 ± 0.01*b*
		120	5890 ± 190*a*	30500 ± 690*b*	0.19 ± 0.01*a*	0.81 ± 0.01*a*	0.46 ± 0.02*b*	0.37 ± 0.02*b*	0.27 ± 0.01*a*	0.10 ± 0.01*b*
	***A. halleri***	0	6800 ± 340*b*	34200 ± 640*a*	0.20 ± 0.01*b*	0.80 ± 0.01*a*	0.51 ± 0.03*a*	0.40 ± 0.02*a*	0.26 ± 0.01*ab*	0.11 ± 0.00*a*
		24	6330 ± 200*b*	33600 ± 680*a*	0.19 ± 0.00*b*	0.81 ± 0.00*a*	0.40 ± 0.02*b*	0.33 ± 0.02*ab*	0.31 ± 0.01*a*	0.10 ± 0.01*a*
		48	7770 ± 750*ab*	32500 ± 650*a*	0.22 ± 0.02*ab*	0.78 ± 0.02*ab*	0.35 ± 0.03*bc*	0.28 ± 0.03*bc*	0.28 ± 0.01*a*	0.08 ± 0.01*ab*
		72	7830 ± 310*ab*	33700 ± 1220*a*	0.24 ± 0.02*ab*	0.76 ± 0.02*ab*	0.44 ± 0.04*ab*	0.33 ± 0.03*ab*	0.20 ± 0.01*b*	0.07 ± 0.01*bc*
		96	7770 ± 310*ab*	34100 ± 1280*a*	0.23 ± 0.02*ab*	0.77 ± 0.02*ab*	0.46 ± 0.03*ab*	0.35 ± 0.03*a*	0.21 ± 0.01*b*	0.07 ± 0.01*bc*
		120	9550 ± 850*a*	33100 ± 1600*a*	0.30 ± 0.02*a*	0.70 ± 0.03*b*	0.27 ± 0.03*c*	0.19 ± 0.03*c*	0.21 ± 0.01*b*	0.04 ± 0.01*c*

*Changes in parameters derived from the chlorophyll a fluorescence signal, describing photosynthetic apparatus of A. arenosa and A. halleri in control and under 1.0 mM Cd or 5.0 mM Zn treatement after 0, 24, 48, 72, 96, and 120 h. Values are means ± SE (n = 8). Means followed by the same letter in a column are not significantly different from each other using the HSD test (P ≤ 0.05). Parameters: F_0_ – minimal fluorescence (at t = 0); F_m_, maximal fluorescence; F_v_, maximum variable fluorescence; ϕP_0_, maximum quantum yield of the primary PSII photochemistry; ΨE_0_, probability (at time 0) that a trapped exciton moves an electron into the electron transport chain beyond Q_A_-roba_0_, quantum yield for electron transport from Q_A_-uantum yield for elec_0_, probability with which an electron from the intersystem electron carriers will move to reduce the end acceptors at the PSI acceptor side; ϕR_0_, quantum yield for the reduction of the end electron acceptors at the PSI acceptor side; ϕD_0_, quantum yield (at t = 0) of energy dissipation*.

Parameters describing characteristics of photosynthetic apparatus in general did not significantly change for both species in the control (Table 1). Changes observed in chlorophyll *a* fluorescence kinetics for Cd treated plants were confirmed by the value of maximum quantum efficiency of the PSII (ϕP_0_) which decreased with the trace metal elements (TME) exposure time (Table 1). Parameters such as quantum yield (ϕE_0_) and probability for electron transport from reduced plastoquinone (Q${}_{\text{A}}^{-}$) to plastoquinone (ψE_0_) significantly decreased toward the end of the experiment only in *A. halleri*. Moreover, the quantum yield (ϕR_0_) and probability for the reduction of the end electron acceptors at the PSI acceptor side (δR_0_) were decreased in both species over the duration of Cd treatment. Quantum yield of energy dissipation (ϕD_0_) increased in both species under Cd treatment (Table 1). ϕP_0_ of *A. arenosa* was not affected by the Zn treatment, whereas in *A. halleri* it was significantly lowered at the end of the experiment (Table 1). ϕE_0_ and ψE_0_ in *A. arenosa* were considerably decreased after 1 days, whereas in *A. halleri* the significant decrease in these parameters was observed at the end of experiment (5 days). δR_0_ was not substantially changed over time in both species treated with Zn, whereas ϕR_0_ was significantly lowered after 1 and 3 days of Zn treatment in *A. arenosa* and *A. halleri*, respectively. ϕD_0_ did not change during the Zn treatment, however, in *A. halleri* it significantly increased only after 5 days (Table 1).

**Figure 6:**
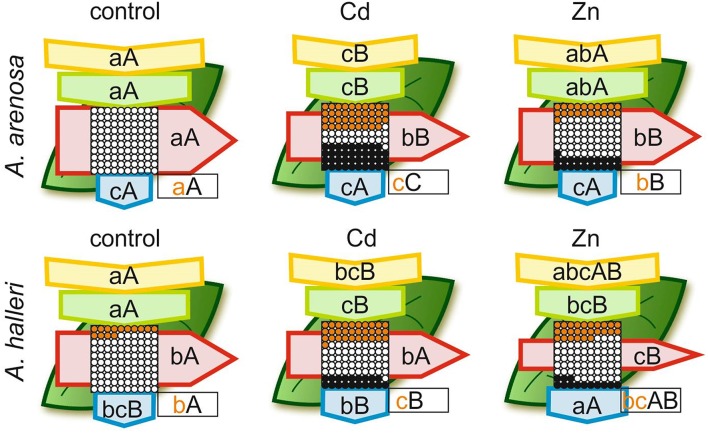
Leaf model showing the phenomenological energy fluxes per the excited cross sections (CS) of the leaves of the *A. arenosa* and *A. halleri* at the end of experiment (120 h). Cd−1.0 mM Cd treatment; Zn−5.0 mM Zn treatment. Each relative value of the measured parameters is the mean (*n* = 20) and the width of each arrow corresponds to the intensity of the flux. Yellow arrow—ABS/CS, absorption flux per CS approximated; green arrow—TR/CS, trapped energy flux per CS; red arrow—ET/CS, electron transport flux per CS; blue arrow—DI/CS, dissipated energy flux per CS; circles inscribed in squares—RC/CS, % of active/inactive reaction centers. White circles inscribed in squares represent reduced Q_A_ reaction centers (active), black (or orange) circles represent non-reduced Q_A_ reaction centers (inactive), 100% of the active reaction centers responded with the highest mean value observed in the control conditions. Orange circles present the difference in % of inactive RC between *A. arenosa* and *A*. halleri. Means followed by the same letter for each parameter in a row (upper case letters) or in a column (lower case letters) are not significantly different from each other using the Tukey HSD test (*P* < 0.05). Letters are inscribed into arrows, except for RC/CS, where they are placed in a box in the bottom right corner of the square with circles. Lower case letters describe statistical differences between species in all treatments, upper case letters describe changes for each species during experiment. Orange lower case letters describe statistical differences in RC/CS between species under each HM treatment.

Overall efficiency of PSII, showed as models of phenomenological energy fluxes per cross section (CS) of *A. arenosa* and *A. halleri*, was differently affected by Cd or Zn treatment after 5 days compared with the control (Figure 6). After 5 days of Cd treatment absorption flux (ABS/CS) was considerably lower in both species compared with the control, whereas Zn treatment did not cause a significant change in both species (Figure 6). Trapped energy flux was significantly diminished by Cd and Zn treatment in both species, except in *A. arenosa* treated with Zn. Electron transport flux (ET/CS) was not lowered compared with the control only in *A. halleri* treated with Cd. Both Cd and Zn treatment caused significant increase in percentage of inactive reaction centers (RC) in *A. arenosa*, whereas in *A. halleri* significant increase in this parameter was observed only in Cd treatment (Figure 6).

### Gas-Exchange Parameters

**Figure 7:**
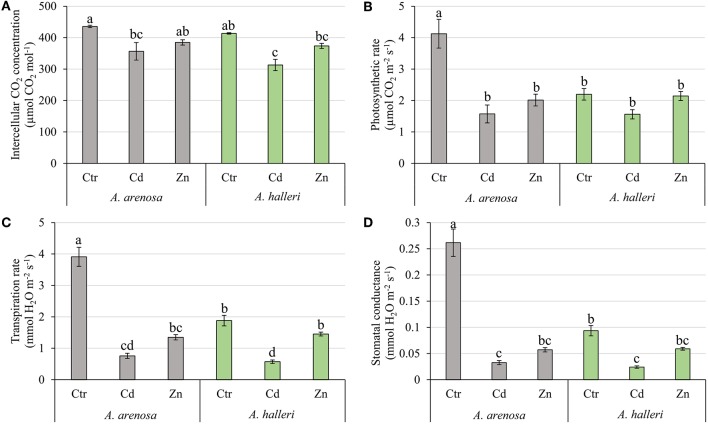
Intercellular CO_2_ concentration **(A)**, Photosynthetic rate **(B)**, Transpiration rate **(C)**, and Stomatal conductance **(D)** in *A. arenosa* (gray) and *A. halleri* (green) after 120 h of treatment. Ctr—control; Cd−1.0 mM Cd treatment; Zn−5.0 mM Zn treatment. Values are means ± SE (*n* = 20). Means followed by the same letter are not significantly different from each other using the HSD test (*P* < 0.05).

The concentration of intercellular CO_2_ (*C*_*i*_) was considerably decreased by 5 days Cd treatment in *A. arenosa* and *A. halleri* (Figure 7A). In control conditions the photosynthetic rate (*A*) of *A. arenosa* was more than twice that of *A. halleri* (Figure 7B). Both Cd ad Zn treatments caused a considerable decrease of the photosynthetic rate only in *A. arenosa* (38 and 49% of control for Cd and Zn, respectively) after 5 days (Figure 7B). In control conditions the transpiration rate (*E*) of *A. arenosa* was twice that of *A. halleri* (Figure 7C). Cd treatment caused a significant decrease in *E* for both species (19 and 30% of the control for *A. arenosa* and *A. halleri*, respectively). On the contrary, *E* significantly decreased during Zn treatment only in *A. arenosa* (35% of the control) (Figure 7C). In the control conditions, the stomatal conductance (*g*_*s*_) of *A. arenosa* was more than twice that of *A. halleri* (Figure 7D). A significant decrease in *g*_*s*_ was observed in Cd treated *A. arenosa* (12% of the control) and *A. halleri* (26% of the control). In Zn treated plants, significant decrease in *g*_*s*_ was observed only in *A. arenosa* (22% of the control) (Figure 7D). All gas-exchange parameters were considerably affected by both treatments and species. Moreover, interaction between treatment type and species was not substantial only for *C*_*i*_ (Table S1).

## Discussion

In order to gain a better understanding of mechanisms underlying metal tolerance of *A. halleri* and *A. arenosa*, metal content, photosynthetic activity, levels of oxidative stress, gas-exchange parameters, chlorophyll, flavonol, and anthocyanin indices were analyzed in metallicolous populations from the same contaminated site in southern Poland grown in control, and exposed to high Cd and Zn concentrations.

Cd and Zn contents in leaves (Figures 1A,B) suggest that the two species differ in the mechanisms involved in the uptake and transport of these metals, with a clear preference for Zn accumulation in *A. halleri* and for Cd accumulation in *A. arenosa*. Since there are no reports, which compare both plant species in control conditions it is impossible to explain these differences. However, it is tempting to suggest that the higher accumulation of Zn by *A. halleri* and higher accumulation of Cd by *A. arenosa* is connected with different transport activity of transporters responsible for the uptake and translocation of both metals in plants. In order to elucidate this deference between the species further research on gene expression levels are needed. Nevertheless, our results showed that the studied metallicolous populations of *A. arenosa* and *A. halleri* are capable of accumulating high concentrations of Cd and Zn. Moreover, the concentrations of Cd and Zn in *A. arenosa* observed in the current study are comparable to the concentrations reported in hyperaccumulator plant species by other authors (Meyer et al., 2010; Farinati et al., 2011; Corso et al., 2018; Schvartzman et al., 2018).

We observed that high Cd treatment (1.0 mM) decreased the growth of shoots in both *A. arenosa* and *A. halleri* (Figure 2A). Despite the higher accumulation of Cd in *A. arenosa* (Figure 1A), the decrease in shoot biomass was slightly different (60% of control) than that observed in *A. halleri* (53% of control). This finding shows high tolerance to Cd of *A. arenosa* and *A. halleri*. In plants treated with high Zn concentration (5.0 mM), the shoot biomass was considerably lowered only in *A. halleri*, but it is important to note that accumulation of Zn in *A. halleri* was almost three-times higher than in *A. arenosa* (Figure 1B).

Our results showed that Cd caused similar and significant increase in the concentrations of hydrogen peroxide and MDA, while Zn treatment did not cause any change compared to the control conditions in *A. arenosa* and *A. halleri* (Figures 3A,B). However, considering contrasting accumulation of Cd and Zn in both species, our data suggests that anti-oxidative mechanisms in *A. arenosa* can better cope with Cd-induced oxidative stress compared to *A. halleri*, while the opposite trend was observed for Zn. Furthermore, large difference in catalase activity in the response to Cd or Zn treatment was found between *A. arenosa* and *A. halleri*, which suggests that various anti-oxidative mechanisms may function in both species. Cd induced oxidative stress in *A. halleri* has been already reported by other authors (Baliardini et al., 2015; Ahmadi et al., 2018).

Measurements of chlorophyll content can be useful for assessing plant tolerance to stress (Rong-hua et al., 2006; Ramirez et al., 2014; Xue et al., 2018). Relatively low alterations in chlorophyll content measured after exposure to high concentrations of the metals (1 mM Cd and 5 mM Zn) suggest that *A. arenosa* is as tolerant to Cd and Zn excess as *A. halleri*, which was not reported so far (Figure 4A). In contrast, Paunov et al. (2018) reported a substantial decrease in chlorophyll content in leaves of durum wheat after 7 days treatment with only 50 μM Cd and 600 μM Zn. Küpper et al. (2007) also showed a significantly higher decrease in chlorophyll content in non-hyperaccumulator *Thlaspi fendleri* after exposure to Cd when compared with *Noccaea caerulescens*.

It was documented in the current study that flavonol and anthocyanin content was constitutively higher in *A. halleri* than in *A. arenosa* (Figures 4B,C). Generally, the content of flavonols lowered in both species after exposure to Cd or Zn, whereas the content of anthocyanins increased. The role of flavonols and anthocyanins during metallic stress has been poorly investigated in plants so far. Nonetheless, Corso et al. (2018) showed recently that flavonols and anthocyanins play an important role in plant response to TME stress in hyperaccumulating and non-hyperaccumulating plant species, most likely through the enhancement of antioxidant capacity and/or metal chelation in plants (Corso et al., 2018). Our results may suggest that *A. arenosa* and *A. halleri* favor biosynthesis of anthocyanins over flavonols in response to the toxic effect of Cd or Zn (Figures 4B,C). However, it is possible that flavonols made the chelates with Cd and Zn and were not detected by the method used in the current study. Such lower detectability of flavonols by fluorometric methods, as a result of chelation with TME, was documented by Kasprzak et al. (2015). Hence, the role of flavonols in response to Cd and Zn toxicity in both plant species should be further investigated.

There is a dearth of data focusing on toxic effects of Cd and Zn on photosynthesis presented as chlorophyll *a* induction transients in metal hyperaccumulator and hypertolerant species such as *A. halleri*, moreover, there are no such reports for *A. arenosa*. Our data showed the presence of ΔK, ΔJ, ΔI, ΔH, and ΔG peaks on ΔV_t_ curves. Positive peaks on ΔV_t_ curves can be linked to the toxic effect of TME on different components of photosynthetic apparatus, whereas negative values might suggest stimulatory effect compared with the control. Under 1.0 mM Cd treatment in *A. arenosa*, which may suggest damage to the OEC (positive ΔK peak), electron transport between Q_A_ and Q_B_ (ΔJ) and connectivity between PSII and PSI, as well as the end electron acceptors of PSI like FNR (ΔI, ΔH, and ΔG) (Figure 5A) (Kalaji and Loboda, 2007; Paunov et al., 2018). On the other hand, in *A. halleri* that accumulated significantly less Cd (Figure 1A), we observed a stimulation of most of components of electron transport chain compared with the control (Figure 5B). Our research for the first time shows such stimulatory effect of Cd on photosynthetic apparatus of *A. halleri*. However, Tang et al. (2016) reported increase in plant growth as well as induction of some photosynthetic parameters (e.g., ϕP_0_) and up-regulation of genes involved in photosynthesis in hyperaccumulator *Sedum alfredii* exposed to low Cd concentration (5 μM). Moreover, similar stimulation of growth of another hyperaccumulator *Noccea caerulescens* was reported by Lombi et al. (2000) that was exposed to Cd (100 μM). By contrast, Paunov et al. (2018) observed a presence of positive ΔK and ΔJ, and negative ΔI and ΔH peaks in ΔV_t_ curve for durum wheat plants treated with 50 μM of Cd for 7 days, whereas Kalaji and Loboda (2007) and Kalaji et al. (2018) reported the complete flattening of chlorophyll *a* fluorescence induction curve for barley treated with only 25 μM Cd for 24 h, which suggested lethal effect of investigated treatment.

Little information is available on the toxic effect of Zn on chlorophyll *a* fluorescence induction curves in plants and PSII functionality under Zn stress (Paunov et al., 2018; Moustakas et al., 2019). Paunov et al. (2018) showed the toxic effect of high Zn (600 μM) treatment, visible as a presence of positive ΔK and ΔJ peaks on chlorophyll *a* fluorescence induction transient in durum wheat. In current research we used 8-fold higher Zn concentration than that used by Paunov et al. (2018) and we showed that OEC (positive ΔK peak), Q_A_ pool (positive ΔJ peak) and PSI components (positive ΔI, ΔH, and ΔG peak) were more affected in *A. arenosa* (Figure 5C) compared with *A. halleri* (Figure 5D) despite higher Zn accumulation in leaves of *A. halleri* (Figure 1B). It should be stressed, however, that the Zn concertation used in the present study was 8-fold higher compared to the concentration used by Paunov et al. (2018).

Our results show that both Cd and Zn have contrasting effects on photosynthetic apparatus of *A. arenosa* and *A. halleri*. Based on presented chlorophyll *a* fluorescence induction curves and available literature we can conclude that photosynthetic apparatus of metallicolous *A. arenosa* population is hypertolerant to Cd and Zn stresses. Although Przedpełska and Wierzbicka (2007) reported that metallicolous population of *A. arenosa* are more tolerant to Cd and Zn than non-metallicolous populations, they did not compare them with any well-known hyperaccumulator plant of both metals.

Maximum quantum efficiency of the PSII (ϕP_0_), otherwise known as F_v_/F_m_, is the most commonly used parameter which describes the state of photosynthetic apparatus, and many reports show its decrease in various plant species under Cd (Huang et al., 2017 Paunov et al., 2018; Xue et al., 2018) or Zn stress treatment (Vassilev et al., 2011; Santos et al., 2014; Paunov et al., 2018). Our results showed that Cd treatment caused ϕP_0_ of *A. arenosa* and *A. halleri* to decrease near the end of experiment compared with the control plants, however, considering extremely high Cd concentration the overall physiological status of both species was not significantly reduced. Moreover, ψE_0_ and ϕE_0_ that are associated with O-J phase were not considerably affected by Cd treatment in both species (Table 1). In this report, exposure for 5 days to 5 mM Zn did not affect ϕP_0_ of *A. arenosa*, whereas in *A. halleri* significant decrease was observed only at the end of experiment (Table 1). Other reports showing decreasing values of ϕP_0_ in *A. arenosa, A. halleri* and *Noccea caerulescens* under Zn treatment (from 10 μM to 3.0 mM) are in good agreement with hypertolerance of photosynthetic apparatus of *A. arenosa* and *A. halleri* presented in the current study (Table 1) (Cho et al., 2003; Küpper et al., 2007; Przedpelska-Wasowicz and Wasowicz, 2013). Kalaji and Loboda (2007) reported that energy fluxes per excited cross section in two cultivars of *Hordeum vulgare* were significantly disrupted after 24 h treatment with 25 μM Cd. In their research in both cultivars, almost all RC were deactivated by Cd and DI/CS was extremely higher compared to the control, causing ET/CS to be almost completely inhibited. The current study shows that 5 days treatment with 1 mM Cd or 5 mM Zn caused in both species that most energy fluxes per excited cross section, although significantly lowered compared with the control, had relatively high values and most of RC were still active (Figure 6). These results suggest that PSII of both species is hypertolerant to high concentration of Cd and Zn. For the first time, we show complete analysis of photosynthetic apparatus (Table 1) and phenomenological energy fluxes per excited cross section (Figure 6) for *A. arenosa* and *A. halleri* treated with high concentration of Cd and excess Zn in controlled conditions.

Our data showed that the studied metallicolous population of *A. arenosa* was extremely tolerant to Cd and Zn, similarly to the Zn, Cd hyperaccumulating *A. halleri* metallicolous population. This study also highlighted contrasting responses of plants growing at the same contaminated site to Cd and Zn treatments, in particular in metal accumulation, photosynthetic parameters [e.g., quantum yield (ϕP_0_)], gas-exchange parameters (e.g., photosynthetic rate) as well as in the content of chlorophyll, flavonols, and anthocyanins.

## Author Contributions

MS, KS, NV, and EM conceived and designed the research. MS, KS, and ŻG conducted the experiments. MS, KS, SR, MC, and CH analyzed the data. MS wrote the first draft of the manuscript, which was edited by all the authors.

### Conflict of Interest Statement

The authors declare that the research was conducted in the absence of any commercial or financial relationships that could be construed as a potential conflict of interest.
