# Supplementary material for: Toxic Effects of Cd and Zn on the Photosynthetic Apparatus of the Arabidopsis halleri and Arabidopsis arenosa Pseudo-Metallophytes
Source: Front Plant Sci. 2019 Jun 6;10:748. doi: 10.3389/fpls.2019.00748 (PMC6563759; doi:10.3389/fpls.2019.00748)
Supplement: Supplementary file 1 [file Table_1.DOCX]

| Table S1 Summary of two-way ANOVA analysis for effect of treatment, plant species and interaction between these two factors on different physiological parameters | | | | | | | | | | | | | |
| --- | --- | --- | --- | --- | --- | --- | --- | --- | --- | --- | --- | --- | --- |
| Factors | Shoot biomass | Root biomass | Root length | H_2_O_2_ concentration | MDA content | Catalase activity | Chlorophyll index | Flavonol index | Anthocyanin index | Intracellular CO_2_ concentration | Photosynthetic rate | Stomatal conductance | Transpiration rate |
| Species | X | X | **−** | **−** | X | X | X | X | **−** | X | X | X | X |
| Treatment | X | X | X | X | X | X | X | X | X | X | X | X | X |
| Species/  treatment | **−** | X | **−** | **−** | **−** | X | **−** | X | X | **−** | X | X | X |

“Species” (*A. arenosa*, *A. halleri*); “Treatment” (Control, 1.0 mM Cd, 5.0 mM Zn); “Species/treatment” **−** interaction between these two factors. “X” means that the factors have statistically significant effect on a measured parameter (*p* <0.05).
