# Supplementary material for: Toxic Effects of Cd and Zn on the Photosynthetic Apparatus of the Arabidopsis halleri and Arabidopsis arenosa Pseudo-Metallophytes
Source: Front Plant Sci. 2019 Jun 6;10:748. doi: 10.3389/fpls.2019.00748 (PMC6563759; doi:10.3389/fpls.2019.00748)
Supplement: Supplementary file 2 [file Table_2.DOCX]

| **Table S2.** Abbreviations and definitions of photosynthesis parameters | |
| --- | --- |
| General terminology | |
| PSII | Photosystem II |
| PSI | Photosystem I |
| OJIP | Transient of chlorophyll *a* fluorescence rise induced during a dark-to-strong light transition, where O is equivalent to F_0_ and P is equivalent to F_m_ |
| CS | Excited cross section of leaf |
| RC | Reaction center of PSII |
| Q_A_ | Primary quinone electron acceptor of PSII |
| FNR | Ferredoxin-NADP^+^ Reductase |
| OEC | Oxygen Evolving Complex |
| MDA | Malondialdehyde |
| CAT | Catalase |
| Fluorescence parameters | |
| F_0_ | Minimal fluorescence, when all PSII RCs are open (at t = 0) |
| F_m_ | Maximal fluorescence, when all PSII RCs are closed |
| F_v_ = F_m_-F_0_ | Maximal variable fluorescence |
| F_t_ | Fluorescence at time t |
| V_t_ = (F_t_-F_0_)/(F_m_-F_0_)  V_tF_ | Relative variable fluorescence at time t  Relative variable fluorescence at time t for control |
| ΔV_t_ = (F_t_-F_0_)/(F_m_-F_0_) - V_tF_ | = V_t_ - V_tF_ |
| Yields or flux ratios | |
| φD_0_ = F_0_/F_m_ | Quantum yield (at t = 0) of energy dissipation |
| φP_0_ = [1-(F_0_/F_m_)] | Maximum quantum yield of primary photochemistry (at t = 0) |
| ΨE_0_ = (1-V_J_) | Probability (at t = 0) that a trapped excition moves an electron into the electron transport chain beyond Q_A_ |
| φE_0_ = [1-(F_0_/F_m_)](1-V_J_) | Quantum yield of electron transport (at t = 0) |
| δR_0_ = (1-V_I_)/(1-V_J_) | Probability with which an electron from the intersystem electron carriers will move to reduce the end acceptors at the PSI acceptor side |
| φR_0_ = (1-F_0_/F_m_)(1-V_I_) | Quantum yield for reduction of end electron acceptors at the PSI acceptor side |
| Phenomenological energy fluxes (per excited cross section of leaf) | |
| ABS/CS | Absorption flux pre CS |
| TR/CS | Trapped energy flux per CS |
| ET/CS | Electron transport per CS |
| DI/CS | Dissipation energy flux per CS |
| RC/CS | % of active reaction centers per CS in comparison with initial state |
| Gas exchange parameters |  |
| A | Photosynthesis rate |
| C_i_ | Intracellular CO_2_ concentration |
| g_s_ | Stomatal conductance |
| E | Transpiration rate |
