# Supplementary material for: Toxic Effects of Cd and Zn on the Photosynthetic Apparatus of the Arabidopsis halleri and Arabidopsis arenosa Pseudo-Metallophytes
Source: Front Plant Sci. 2019 Jun 6;10:748. doi: 10.3389/fpls.2019.00748 (PMC6563759; doi:10.3389/fpls.2019.00748)
Supplement: Supplementary file 3 [file Data_Sheet_1.PDF]

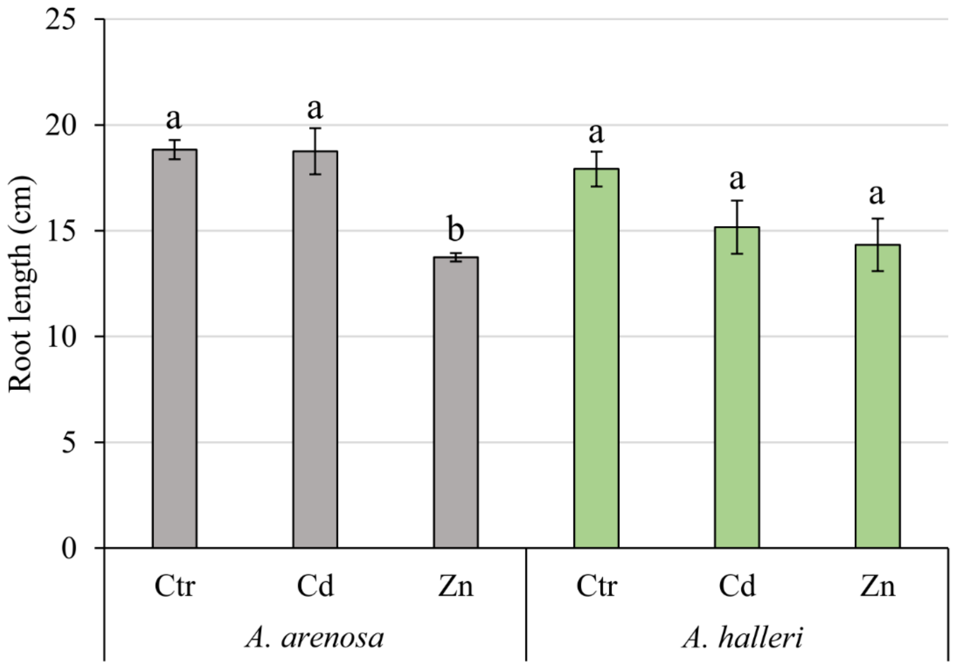

Figure S1. Length of roots of *A. arenosa* (grey) and *A. halleri* (green) at the end of experiment (120 h). Ctr – control; Cd – 1.0 mM Cd treatment; Zn – 5.0 mM Zn treatment. Values are means ± SE (n = 6). Means followed by the same letter for each species are not significantly different from each other using the HSD test ($P < 0.05$).
