# Supplementary material for: Toxic Effects of Cd and Zn on the Photosynthetic Apparatus of the Arabidopsis halleri and Arabidopsis arenosa Pseudo-Metallophytes
Source: Front Plant Sci. 2019 Jun 6;10:748. doi: 10.3389/fpls.2019.00748 (PMC6563759; doi:10.3389/fpls.2019.00748)
Supplement: Supplementary file 4 [file Data_Sheet_2.PDF]

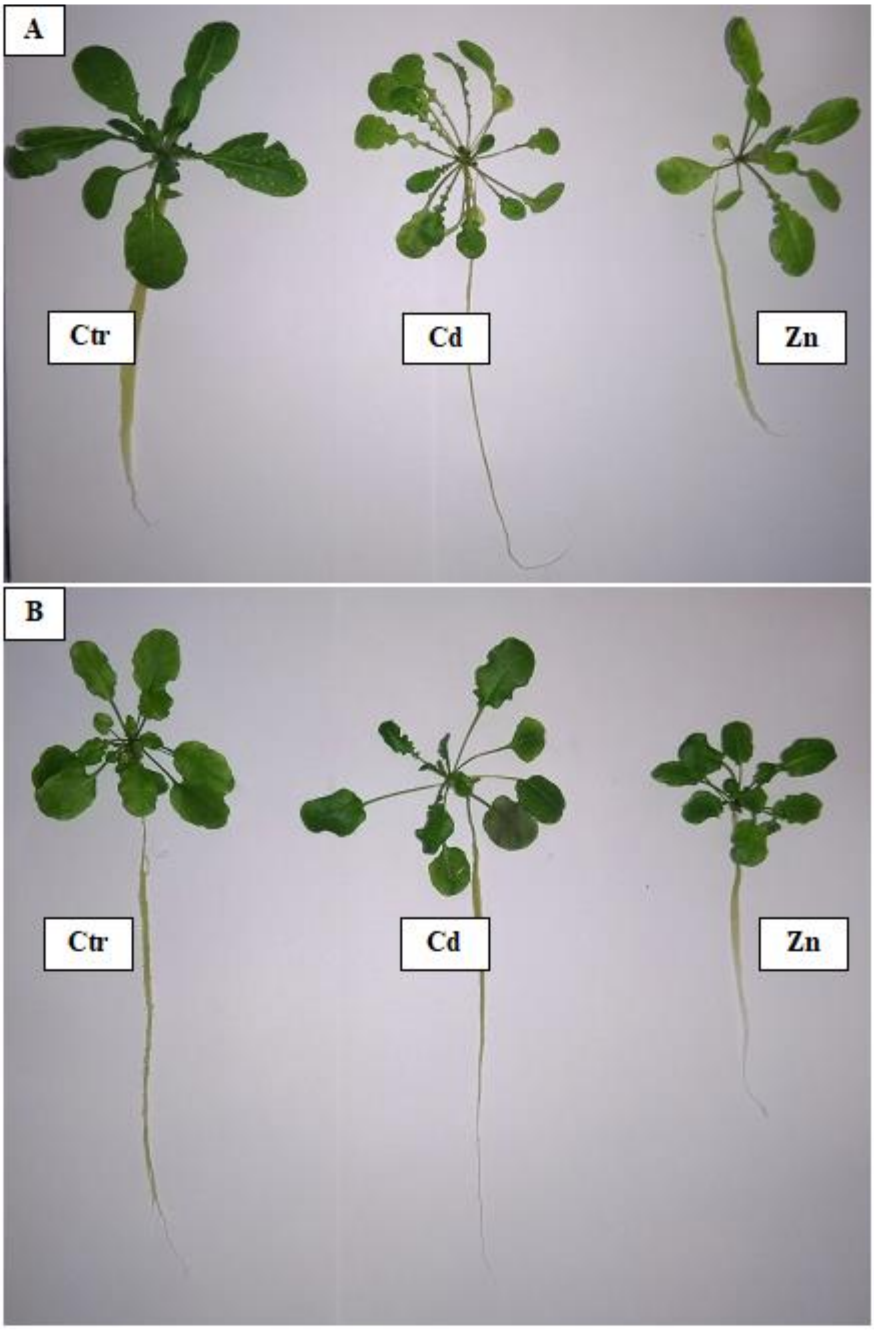

Figure S2. The pictures of *A. arenosa* (A) and *A. halleri* (B) at the end of experiment; Ctr – control; Cd – 1.0 mM Cd treatment; Zn – 5.0 mM Zn treatment.
