# Supplementary material for: Toxic Effects of Cd and Zn on the Photosynthetic Apparatus of the Arabidopsis halleri and Arabidopsis arenosa Pseudo-Metallophytes
Source: Front Plant Sci. 2019 Jun 6;10:748. doi: 10.3389/fpls.2019.00748 (PMC6563759; doi:10.3389/fpls.2019.00748)
Supplement: Supplementary file 5 [file Data_Sheet_3.PDF]

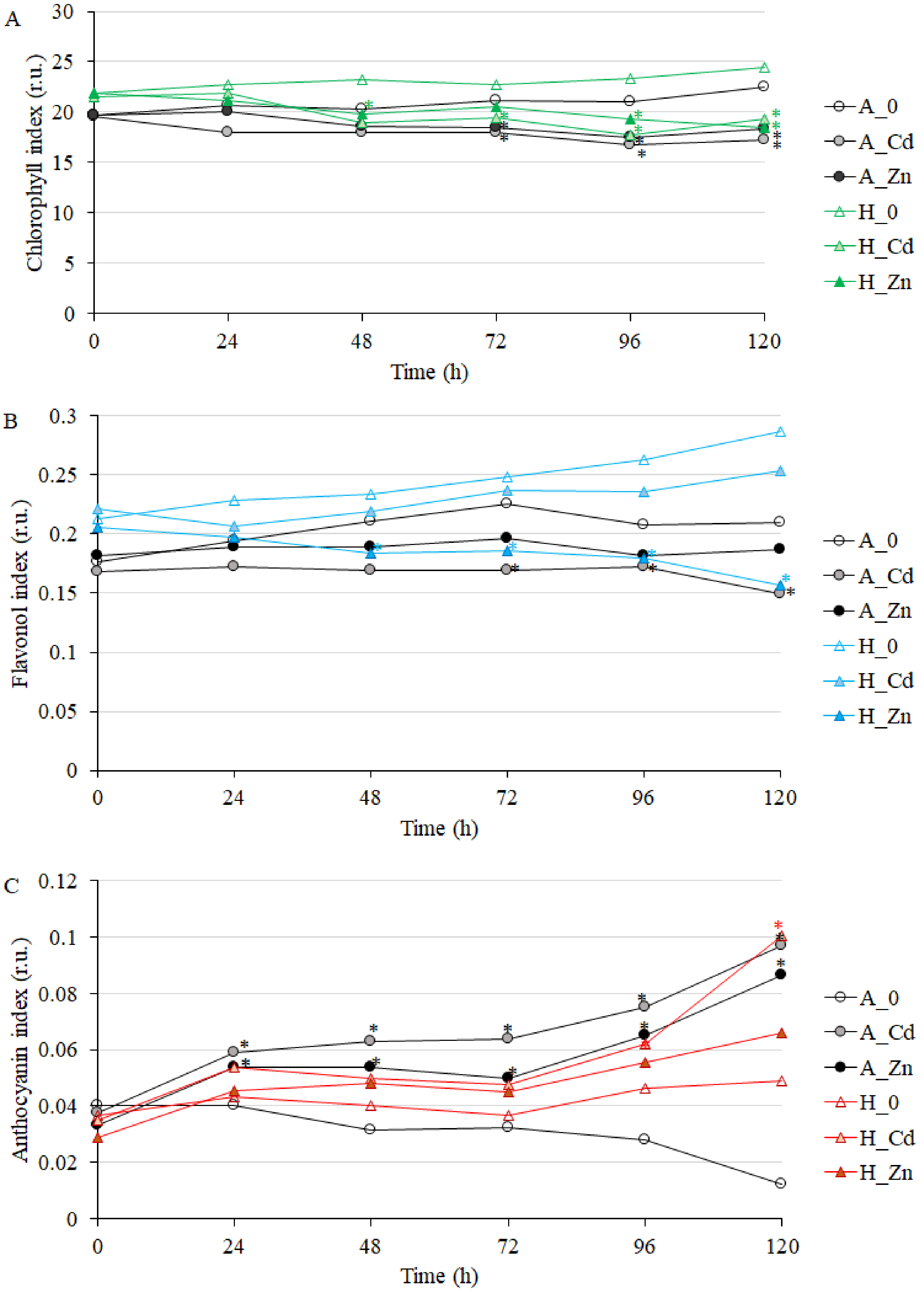

Figure S3. Changes in chlorophyll (A), flavonol (B) and anthocyanin (C) index (relative units) in *A. arenosa* (A_treatment) and *A. halleri* (H_treatment) leaves in control and under Cd or Zn treatment during 120 hours. Values are means (n = 30). Asterisk (*) means significant difference compared to control using Tukey HSD test ($P < 0.05$).
